# Supplementary material for: Indocyanine green fluorescence lymphography: An exploratory study of superficial lymphatic territories in the head and hind limbs of 33 cat cadavers
Source: PLoS One. 2025 Jun 30;20(6):e0327005. doi: 10.1371/journal.pone.0327005 (PMC12208428; doi:10.1371/journal.pone.0327005)
Supplement: S1 Table — LC = Lymphocenter; *, ** = those interrupted lymphatic pathways were detected on the same cadaver, respectively. (PDF) [file pone.0327005.s001.pdf]

**Selected Cutaneous region**	**Detected draining LC**	**Description of interrupted lymphatic pathway**
Rostral mandibular	Mandibular	Medial to the principal lymphatic drainage root
Rostral maxillary*	Mandibular	Run parallel to the principal pathway, interrupted few millimeters after detection
Lateral thigh - cranial	Superficial inguinal	Medial aspect of the genicular region, progression of 1 cm length distal to the injected area, towards popliteal lymph node
Medial Genicular	None	Progression 1 cm distal to the injected area
Medial Crural	None	Progression of 2 cm directed towards superficial inguinal LC
Lateral Tarsal	Popliteal	Medial to the genicular region
Lateral Tarsal **	Popliteal	Progression of 1 cm directed versus the inguinal LC
Medial Tarsal **	None	Progression of 1 cm directed versus the inguinal LC
Dorsal Metatarsal	Popliteal	Medial surface of the foot, progressed proximally on the medial aspect of tibial region, interrupted at the level of the knee
Dorsal Metatarsal *	Popliteal	Run parallel to the principal pathway, interrupted at the distal third of the tibial region

Dorsal Metatarsal	Popliteal	Progression proximal to popliteal LC, deepened in the distal cranial femoral region
Dorsal Phalangeal	Popliteal	Visible from injected region until the caudal femoral region, then deepened and got lost
Plantar Phalangeal	None	From injected region to the distal aspect of tibial region
