# Supplementary material for: Indocyanine green fluorescence lymphography: An exploratory study of superficial lymphatic territories in the head and hind limbs of 33 cat cadavers
Source: PLoS One. 2025 Jun 30;20(6):e0327005. doi: 10.1371/journal.pone.0327005 (PMC12208428; doi:10.1371/journal.pone.0327005)
Supplement: S2 Table — Only successful lymphographies were considered (21/26 head, 42/58 hind limb). LC = Lymphocentrum. In 1/63 NIRF-ICG lymphography, the detected LC (right mandibular) was contralateral to the selected cutaneous region side (left rostral mandibular). (PDF) [file pone.0327005.s002.pdf]

| | **Head (21)** | **Hind Limb (42)** | **Overall (63)** |
|---|---|---|---|
| **Correspondence with canine lymphosomes (Suami 2013)** | | | |
| Total | 13/21 (62%) | 15/42 (36%) | 28/63 (44%) |
| Partial | 7/21 (33%) | 15/42 (36%) | 22/63 (35%) |
| Non-correspondent | 1/21 (5%) | 12/42 (28%) | 13/63 (21%) |
| **LC** | | | |
| Single | 14/21 (67%) | 33/42 (79%) | 47/63 (75%) |
| Multiple | 7/21 (33%) | 9/42 (21%) | 16/63 (25%) |
| **Detected LC** | 18 Mandibular<br>7 Parotid<br>3 superficial cervical<br>3 medial retropharyngeal<br>**Total 31** | 8 medial iliac<br>10 superficial inguinal<br>33 popliteal<br>**Total 51** | **Total 82** |
| **Shine through effect (LC)** | 3/31 (10%) | 13/51 (25%) | 16/82 (19.5%) |
| **LN per LC** | | | |
| Single | 15/31 (48%) | 44/51 (86%) | 59/82 (72%) |
| Multiple | 16/31 (52%) | 7/51 (14%) | 23/82 (28%) |
