# Supplementary material for: Indocyanine green fluorescence lymphography: An exploratory study of superficial lymphatic territories in the head and hind limbs of 33 cat cadavers
Source: PLoS One. 2025 Jun 30;20(6):e0327005. doi: 10.1371/journal.pone.0327005 (PMC12208428; doi:10.1371/journal.pone.0327005)
Supplement: S3 Table — BCS = Body Condition Score. Statistical association: P < 0.05. (PDF) [file pone.0327005.s003.pdf]

**Variable**<br><br>**TOTAL**	**NIRF-ICG LYMPHOGRAPHY**<br>**Failed**<br>**21/84**	 **Successful**<br>**63/84**
**BCS class**		
Low BCS (46)	11/84	35/84
Normal-High BCS (38)	10/84	28/84
	P=0.80	
**Storage condition**		
Frozen (69)	18/84	51/84
Refrigerated (15)	3/84	12/84
	P=0.75	
**Anatomical district**		
Head (26)	5/84	21/84
Hind limb (58)	16/84	42/84
	P=0.41	
**Selected cutaneous region**		
Auricular (5)	4/84*	1/84
Rostral mandibular (4)	0/84	4/84
Aboral mandibular (4)	0/84	4/84
Rostral maxillary (4)	0/84	4/84
Aboral maxillary (4)	0/84	4/84
Temporal-Zygomatic (5)	1/84	4/84
Lateral thigh – cranial (4)	1/84	3/84
Lateral thigh – caudal (4)	2/84	2/84
Medial thigh – cranial (4)	0/84	4/84
Medial thigh – caudal (4)	1/84	3/84
Lateral Genicular (4)	1/84	3/84
Medial Genicular (4)	2/84	2/84
Lateral Crural (4)	1/84	3/84
Medial Crural (5)	4/84*	1/84
Lateral Tarsal (4)	0/84	4/84
Medial Tarsal (4)	2/84	2/84
Dorsal Metatarsal (4)	0/84	4/84
Plantar Metatarsal (4)	0/84	4/84
Dorsal Phalangeal (4)	0/84	4/84
Plantar Phalangeal (5)	2/84	3/84
	**P=0.02***	
**Cutaneous pigmentation**		
Yes (10)	2/84	8/84
No (74)	19/84	55/84
	P=0.70	
**N. of multiple injections spots**	Median: 10 (6-16)	Median: 8 (3-20)
	P=0.21	
**Volume injected**		
**Full (71)**	21/84	50/84
**Half (13)**	0/84*	13/84
	**P=0.02***	
